# The arms race between beet necrotic yellow vein virus and host resistance in sugar beet

**DOI:** 10.3389/fpls.2023.1098786

**Published:** 2023-03-31

**Authors:** Sebastian Liebe, Edgar Maiss, Mark Varrelmann

**Affiliations:** ^1^ Department of Phytopathology, Institute of Sugar Beet Research, Göttingen, Germany; ^2^ Department of Phytomedicine, Plant Virology, Institute of Horticultural Production Systems, Leibniz University, Hannover, Germany

**Keywords:** BNYVV, *Rz1*, resistance-breaking, virus evolution, *Rz2*

## Abstract

Beet necrotic yellow vein virus (BNYVV) causes rhizomania disease in sugar beet (*Beta vulgaris*), which is controlled since more than two decades by cultivars harboring the *Rz1* resistance gene. The development of resistance-breaking strains has been favored by a high selection pressure on the soil-borne virus population. Resistance-breaking is associated with mutations at amino acid positions 67-70 (tetrad) in the RNA3 encoded pathogenicity factor P25 and the presence of an additional RNA component (RNA5). However, natural BNYVV populations are highly diverse making investigations on the resistance-breaking mechanism rather difficult. Therefore, we applied a reverse genetic system for BNYVV (A type) to study *Rz1* resistance-breaking by direct agroinoculation of sugar beet seedlings. The bioassay allowed a clear discrimination between susceptible and *Rz1* resistant plants already four weeks after infection, and resistance-breaking was independent of the sugar beet *Rz1* genotype. A comprehensive screen of natural tetrads for resistance-breaking revealed several new mutations allowing BNYVV to overcome *Rz1*. The supplementation of an additional RNA5 encoding the pathogenicity factor P26 allowed virus accumulation in the *Rz1* genotype independent of the P25 tetrad. This suggests the presence of two distinct resistance-breaking mechanisms allowing BNYVV to overcome *Rz1*. Finally, we showed that the resistance-breaking effect of the tetrad and the RNA5 is specific to *Rz1* and has no effect on the stability of the second resistance gene *Rz2*. Consequently, double resistant cultivars (*Rz1*+*Rz2*) should provide effective control of *Rz1* resistance-breaking strains. Our study highlights the flexibility of the viral genome allowing BNYVV to overcome host resistance, which underlines the need for a continuous search for alternative resistance genes.

## Introduction

The rhizomania disease in sugar beet is caused by beet necrotic yellow vein virus (BNYVV), which belongs to the genus *Benyvirus* within the family *Benyviridae* (Gilmer and Ratti, 2017). The disease occurs worldwide leading to a strong reduction of beet yield and sugar content (Henry, 1996; McGrann et al., 2009). Disease symptoms on the taproot are characterized by extensive lateral root proliferation, reduced size and necrosis of vascular tissue. Vein yellowing and necrosis can be observed on leaves after systemic movement, but these symptoms appear only seldom under field conditions. The root infecting plasmodiophoromycete *Polymyxa betae* is responsible for the transmission of BNYVV (Keskin, 1964; Tamada and Kondo, 2013). Infectious virus particles present in resting spores of *P*. *betae* allow the virus to survive in the soil for decades.

The genome of BNYVV comprises four to five positive-sense single-stranded RNAs which possess a capped 5’-end and a 3’ poly(A) tail (Gilmer and Ratti, 2017). The open reading frame (ORF) of RNA1 encodes an RNA-dependent RNA polymerase containing motifs for methyltransferase, helicase and a papain-like protease (Bouzoubaa et al., 1987). The RNA2 harbors the coat protein (CP) gene which is terminated by a leaky UAG stop codon, resulting in translation of a readthrough protein (CP-RT) necessary for vector transmission by *P*. *betae* (Niesbach-Klösgen et al., 1990; Haeberlé et al., 1994; Tamada et al., 1996). The triple gene block proteins 1-3 responsible for cell-to-cell movement are encoded by three independent ORFs downstream of CP-RT (Gilmer et al., 1992). The RNA2 3´ terminal ORF encodes the viral suppressor of gene silencing (Chiba et al., 2013). The pathogenicity factor P25 responsible for rhizomania symptoms in sugar beet is encoded on RNA3 (Tamada et al., 1999). Successful vector transmission by *P*. *betae* is additionally mediated by P31 encoded on RNA4 (Tamada and Abe, 1989). A fifth RNA species (RNA5) harboring one ORF encoding (P26) can be found in certain virus populations of BNYVV (Tamada et al., 1989; Koenig et al., 1997; Miyanishi et al., 1999). P26 is regarded as a second pathogenicity factor sharing significant sequence homology with the RNA3 encoded P25 (Koenig et al., 1997).

Based on their CP sequence, most BNYVV isolates are classified into two major virus types (A and B type) which are worldwide distributed (Kruse et al., 1994). Additionally, there is another minor virus type (P type), which is closely related to the A type (Miyanishi et al., 1999), but displays only a limited distribution mainly within the French region Pithiviers and a few other countries (Koenig et al., 1997; Koenig and Lennefors, 2000; Ward et al., 2007; Mehrvar et al., 2009). P type populations carry an additional RNA5 which shares high sequence homology with the RNA5 species found in Asian A and B type populations (Koenig et al., 1997). Phylogenetic analysis revealed that all RNA5 species fall into three clusters (Miyanishi et al., 1999). Group I and II (J type) comprise RNA5 species found in Asian isolates and group III (P type) contains RNA5 species from French P type isolates. Recently, we found both RNA5 types (J and P) in different European countries suggesting a broader distribution as previously assumed (Liebe and Varrelmann, 2022). The exact function of the RNA5/P26 during virus infection in sugar beet remains elusive, but two recent publications indicate a role in enhanced symptom severity and *Rz1* resistance-breaking (Tamada et al., 2021; Müllender et al., 2022).

The use of resistant sugar beet cultivars is the only control strategy to avoid high yield losses due to BNYVV. The major resistance gene is *Rz1*, which was identified in 1983 and subsequently introduced in all cultivars (Lewellen et al., 1987). This exerted a high selection pressure on the virus population leading to the first report of *Rz1* resistance-breaking isolates (A type) in the US (Liu et al., 2005; Liu and Lewellen, 2007), and later in different European countries (Pferdmenges et al., 2009; Bornemann et al., 2015; Galein et al., 2018; Yilmaz et al., 2018). Comprehensive sequence analysis elucidated a high variability at the amino acid (aa) positions 67-70 (tetrad) in the pathogenicity factor P25 and certain tetrads could be identified only in resistance-breaking populations (Liu and Lewellen, 2007; Acosta-Leal et al., 2010; Chiba et al., 2011; Bornemann et al., 2015). Until now, more than 20 different tetrads have been described in the literature, but the resistance-breaking properties have been only confirmed for AYPR, VLHG and VCHG by means of reverse genetics (Koenig et al., 2009; Liebe et al., 2020). Apart from the tetrad in P25, *Rz1* resistance-breaking is also associated with the presence of RNA5. Natural populations of the P type are resistance-breaking (Pferdmenges et al., 2009; Bornemann and Varrelmann, 2011; Bornemann et al., 2015) and we recently showed by reverse genetics that this is due to the presence of RNA5 (Müllender et al., 2022). A similar resistance-breaking property has been suggested for the J type RNA5 in Asian A-and B type isolates, but experimental evidence by reverse genetics is lacking (Nakagami et al., 2021; Tamada et al., 2021). Interestingly, we found recently in resistance-breaking isolates from European countries both RNA5 species (J-and P type) in association with A- and B type (Liebe and Varrelmann, 2022). This finding further supports the role of RNA5 in resistance-breaking.

Our knowledge on *Rz1* resistance-breaking by BNYVV has increased since the first emergence of populations capable of overcoming host resistance. Previous studies highlighted the genetic diversity of natural BNYVV populations which complicates the identification of resistance-breaking mutations (Schirmer et al., 2005; Chiba et al., 2011; Galein et al., 2018). In contrast, a reverse genetic system based on a full-length clone of BNYVV (A type) enabled us to study the resistance-breaking mechanism in a previous study (Liebe et al., 2020). In this follow-up study, we have refined the reverse genetic system which can now initiate rhizomania disease in sugar beet after agrobacterium-mediated infection and allows a clear differentiation between susceptible and resistant genotypes. This enabled us to perform a comprehensive screen in which we identified mutations and genome components mediating *Rz1* resistance-breaking. We highlight the flexibility of the viral genome allowing BNYVV to overcome *Rz1*, but we also show that the resistance-breaking properties have no effect on the stability of the alternative resistance gene *Rz2*. Our study increases the knowledge on how plant viruses overcome plant resistance traits with the example of BNYVV which is one of the best studied viruses in this research field.

## Material and methods

### Virus inoculation and growing conditions

If not otherwise stated, all infection experiments were done using a susceptible (KWS03) and a *Rz1* resistant (Beta4430) or *Rz1*+*Rz2* resistant (Angelina) sugar beet genotype provided by KWS SAAT SE & Co. KGaA. Further *Rz1* resistant genotypes were provided by MariboHilleshoeg, SESVanderHave and Strube Research GmbH & Co. KG to validate our assay. The number inoculated plants per treatment varied between 12 and 18 as indicated in the individual experiments. *Agrobacterium tumefaciens*-mediated delivery of our BNYVV A type clone was applied to infect sugar beets. Binary plasmids (pDIVA) carrying the different viral RNAs (RNA1-4) of the A type clone (NCBI Acc. No.: KX665536, KX665537, KX665538, and MF476800) as well as newly generated RNAs were transformed into electrocompetent cells of the *Rhizobium radiobacter* (syn. *Agrobacterium tumefaciens*/*Agrobacterium fabrum*) strain C58/C1 (Voinnet et al., 1998). Each bacterial culture carrying a single plasmid with a cDNA of one of the viral RNAs was grown on selective agar media for two days. Before inoculation, a micro-spoon (Carl Roth, Art. No. 6186.1) with a width of 5 mm was used to scrape from each plate a similar amount of bacterial culture (until the spoon is completely filled). The different cultures were then mixed manually in an empty petri dish. The mixture of these cultures contained at least all plasmids coding for the entire set of genomic RNAs required for BNYVV infection (RNA 1-4, RNA5 optional). An insulin needle (BD Micro-Fine™, 0.3 mm needle diameter) was dipped into the culture and subsequently used to puncture 7-9 old sugar beet seedlings at three different positions along the hypocotyl. Inoculated plants were directly covered for 4 days with a plastic bag. Plants were grown for 6 weeks at 24°C (day)/18°C (night), and a 14 h photoperiod of additional light was introduced.

### Virus quantification

The virus content in lateral roots (100–150 mg) of sugar beets was determined with a double antibody sandwich enzyme-linked immunosorbent assay (DAS-ELISA). Root samples from each plant were analyzed individually in the ELISA. Antibodies specific for BNYVV CP (AS-0737) were obtained from the Leibniz Institute DSMZ-German Collection of Microorganisms and Cell Cultures (Braunschweig, Germany) and ELISA was conducted according to the manufacturer’s instructions. All samples were diluted in sample buffer (1:150 wt/wt) and homogenized with the Precellys 24 tissue homogenizer (Bertin Instruments) for 45 s at 5000 rpm. Raw absorbance values measured at 405 nm were corrected by subtraction of blank and buffer control. Only samples with an absorbance value higher than the mean of the healthy control plus three times standard deviation were considered as infected.

### Site-directed mutagenesis of the P25 tetrad

All tetrads tested for resistance-breaking properties were selected from studies which reported their occurrence in natural population of BNYVV (Schirmer et al., 2005; Chiba et al., 2011; Galein et al., 2018; Yilmaz et al., 2018; Liebe and Varrelmann, 2022). Mutagenesis of the P25 tetrad was done by re-amplification of the plasmid pDIVA containing the full-length BNYVV RNA3 cDNA clone with the P25 tetrad ALHG. First, the plasmid was amplified with two different primer pairs resulting in two PCR products with overlapping termini. The tetrad specific forward primer was combined with a general reverse primer “npErev” and the tetrad specific reverse primers was combined with a general forward primer “npEfor” (**Supplementary Table 1**). This strategy was selected as it introduces a *EcoR*I restriction site into the backbone of pDIVA allowing the selection of mutant plasmids after *in vitro* assembly and transformation. All PCRs were carried out with the Phusion Flash High-Fidelity PCR Master Mix (Thermofisher). Both amplified products were gel purified with the NucleoSpin Gel and PCR Clean−up (Macherey-Nagel). The amplified PCR products were joined using Gibson assembly as previously described (Gibson et al., 2009; Laufer et al., 2018). All plasmids were transformed into chemical competent *Escherichia coli* cells of the strain DH5α or NM522 (Hanahan, 1983; Inoue et al., 1990). The mutations were confirmed by commercial capillary Sanger sequencing using specific sequencing primers (**Supplementary Table 1**). Correct plasmids were transformed into *R. radiobacter* strain C58/C1 as described above.

### Replacement of P25 ORF in RNA3 of the A type clone

Gibson assembly was applied for the replacement of the P25 ORF in the RNA3 of the A type clone by a P25 ORF from the P and B type. The P25 ORF from the P type (soil ID 13) and B type (soil ID 26) were obtained from wild type populations collected in a previous study (Liebe and Varrelmann, 2022). For virus isolation, bait plants were grown in infested soil samples and lateral roots (100-150 mg) were harvested and subjected to RNA extraction using the NucleoSpin RNA Plant kit (Macherey-Nagel) as previously described (Liebe and Varrelmann, 2022). Reverse transcription of RNA (500ng) was done with the RevertAid Reverse Transcriptase and oligo(dT)18 primer (Thermofisher). The P25 ORFs from the B and P type were amplified with specific primers (**Supplementary Table 2**) using the Phusion Flash High-Fidelity PCR Master Mix. The primers consisted of a P25 and a cloning vector specific sequence. Additionally, the plasmid containing the RNA3 from the A type clone was reamplified with specific primers in order to remove the entire P25 ORF and to linearize the plasmid. All PCR products were gel purified before cloning. The amplified P25 ORF from P and B type was cloned into the backbone of RNA3 A type clone using Gibson assembly as described above. After assembly, *in vitro* recombination products were transformed into chemical competent *E. coli* cells as described above. Plasmids carrying the RNA3 (*EcoR*I/*Xba*I: 3544bp, 2561 bp) were identified by means of appropriate fast restriction enzyme digest (Thermofisher) and verified by commercial capillary Sanger sequencing (Eurofins MWG Operon). Subsequently cDNA clones were transformed into *R. radiobacter* strain C58/C1. Full-length sequences of both P25 types and were deposited in the database of the National Center for Biotechnology information (NCBI) as accession numbers OP630457 and OP630458.

### Construction of infectious RNA5 cDNA clones

Two different RNA5 species, namely from P and J type, were cloned to study their effect on resistance-breaking. The P type RNA5 was derived from a French population (soil ID 1) and the J type RNA5 was derived from German population (soil ID 2) which have been described in a previous study (Liebe and Varrelmann, 2022). RNA was extracted from virus infected lateral roots and transcribed into cDNA as described above. Each full-length RNA5 was amplified with specific primers (**Supplementary Table 3**) using the Phusion Flash High-Fidelity PCR Master Mix. PCR products were gel purified and Gibson assembly was applied for the cloning of full-length cDNAs under control of 35S promoter from cauliflower mosaic virus into the linearized plasmid pDIVA (Laufer et al., 2018). After transformation into chemical competent *Escherichia coli* cells (strain DH5α), plasmids carrying the RNA5 (*EcoR*I/*Xba*I: 3324 bp, 2137 bp, 220 bp) were identified by means of appropriate fast restriction enzyme digest. Plasmids were further verified by commercial capillary Sanger sequencing. Verified cDNA clones were transformed into *R. radiobacter* strain C58/C1. Full-length sequences of both RNA5s were deposited in the NCBI database as accession numbers OP630455 and OP630456.

### Bioinformatic and statistical analysis

All sequences in this study were analyzed with the MEGA 7.0 software (Kumar et al., 2016). Multiple sequence alignments of P25 and P26 sequences were performed using the ClustalW algorithm (default settings). Data analysis and visualization of ELISA results was done in the statistic software R using the package ggplot (Wickham, 2009; R Core Team, 2017).

## Results

### Establishment of a resistance assay in sugar beet using a BNYVV A type clone

**Figure 1:**
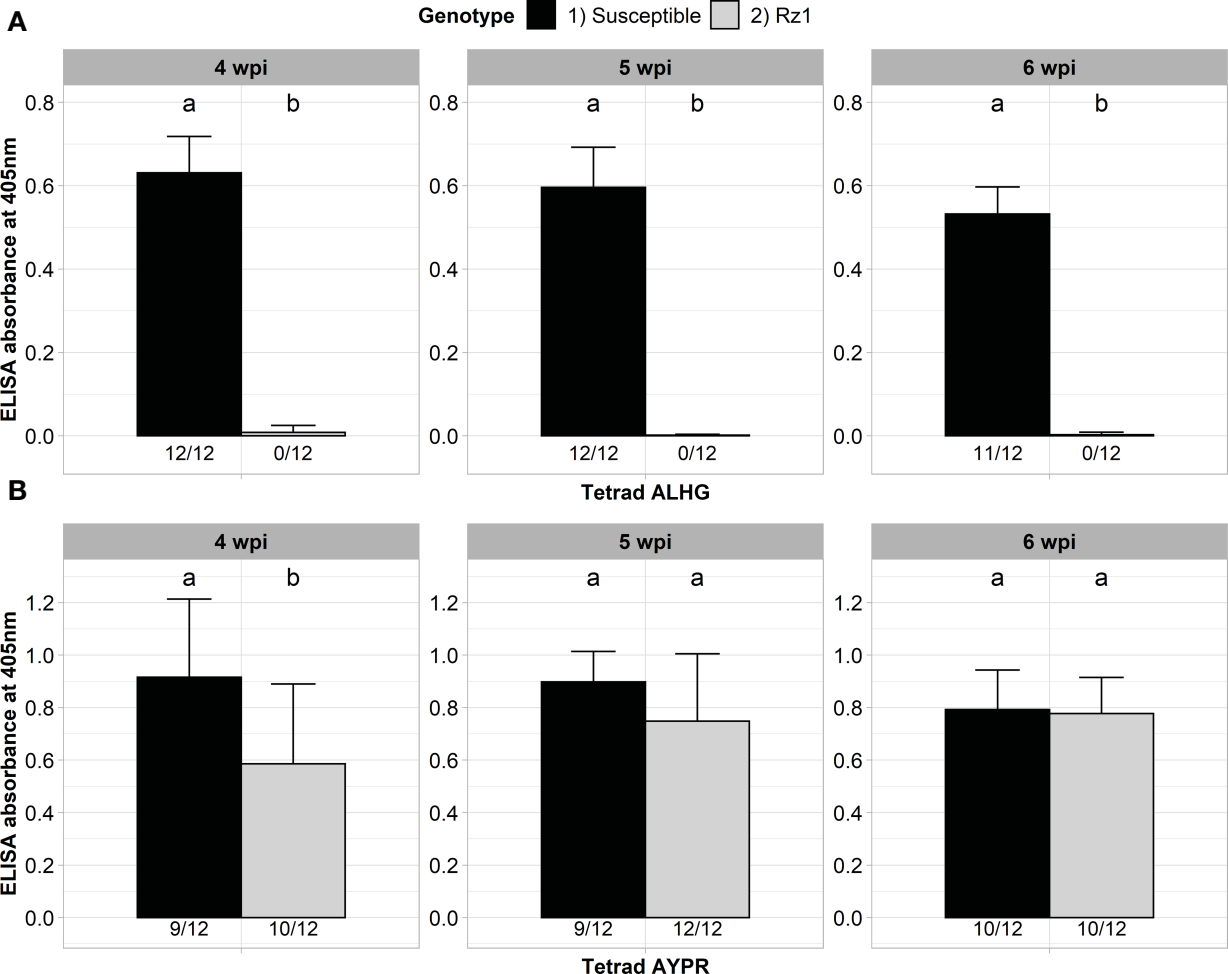
ELISA absorbance values for BNYVV measured in lateral roots of plants from a susceptible and *Rz1* resistant sugar beet genotype (N=12). **(A)** Plants were either inoculated with a non-resistance-breaking (ALHG) or **(B)** with a resistance-breaking (AYPR) BNYVV clone. Lateral roots were harvested 4, 5 and 6 weeks after inoculation (wpi). The numbers below each bar indicate the infection rate (infected/inoculated). Mean values were calculated using only infected plants, whereas all values were averaged when no plant was infected. Error bars indicate standard deviation. The healthy controls are not shown because the absorbance values were zero. Significant differences between both genotypes were determined by the Wilcoxon test and are indicated by small letters (p < 0.05).

Our previously developed BNYVV A type clone was derived from a non-resistance-breaking population with the P25 tetrad ALHG. We previously showed that this clone is not able to accumulate in a *Rz1* genotype after direct agroinoculation of sugar beet seedlings (Müllender et al., 2022). We first confirmed that our clone is not able to infect the *Rz1* genotype after agrobacterium-mediated infection (**Figure 1A**). A susceptible genotype did serve as infection control and the virus titer was measured after 4, 5 and 6 weeks post infection (wpi). The clone could be detected in most inoculated plants of the susceptible genotype already 4 weeks after infection. In contrast, no infection was detectable by ELISA in the *Rz1* genotype at any time point. Statistical significance was confirmed which demonstrates the suitability of our assay to discriminate between both sugar beet genotypes. Subsequently, we performed a proof-of-principle experiment to show that our BNYVV clone can overcome *Rz1* resistance when the P25 tetrad ALHG is replaced by the resistance-breaking tetrad AYPR (**Figure 1B**). The accumulation of the BNYVV clone with AYPR could be confirmed in the susceptible genotype at any time point. Similarly, most of the plants from the *Rz1* genotype were infected and statistical analysis indicated no differences between both genotypes except at the first harvest time point after 4 wpi. Therefore, we could successfully prove that a replacement of the tetrad enabled our clone to overcome *Rz1* resistance after agrobacterium-mediated infection.

**Figure 2:**
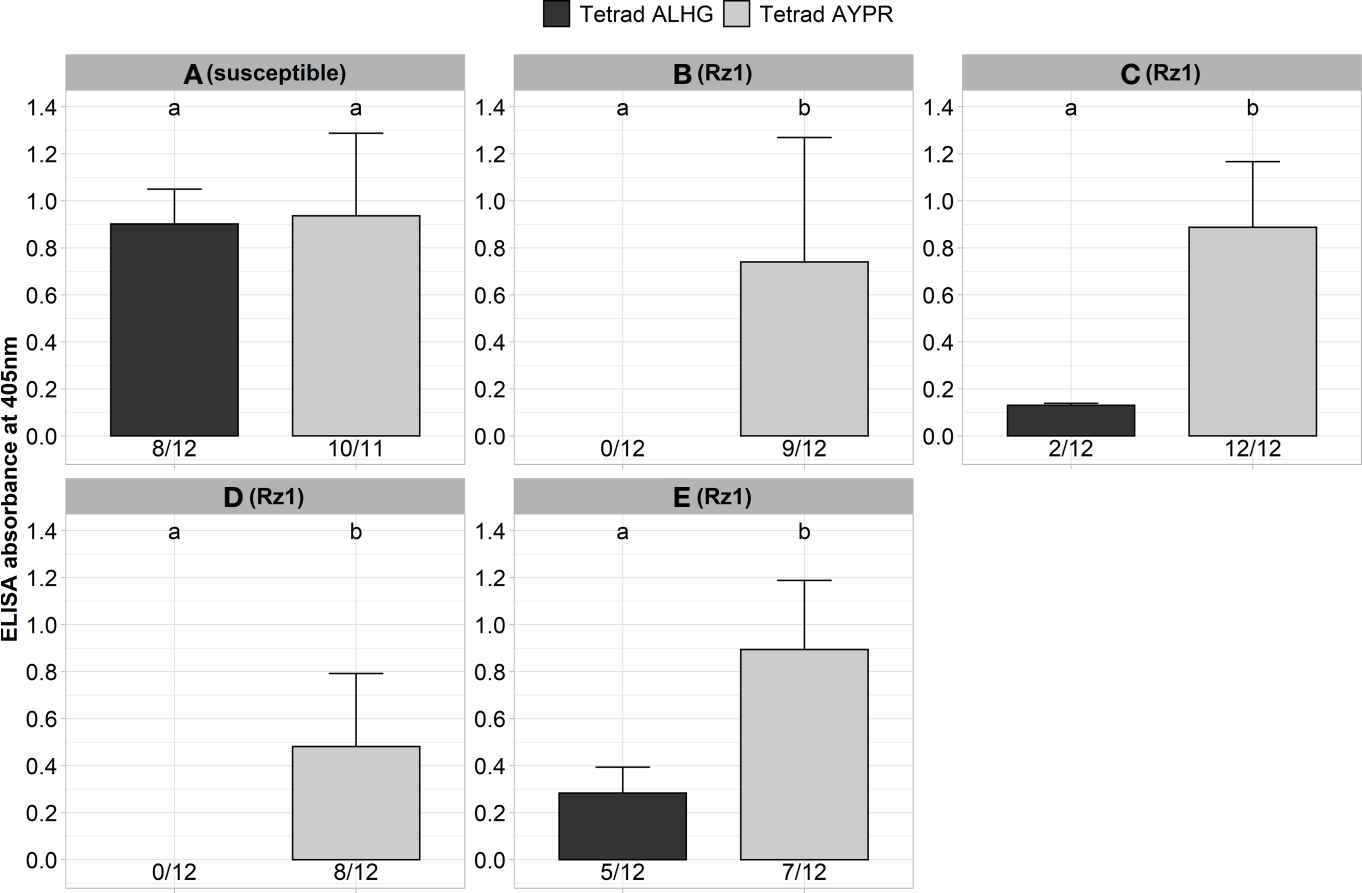
ELISA absorbance values for BNYVV measured in lateral roots of plants from a susceptible **(A)** and different *Rz1* resistant sugar beet genotypes [**B–E**, *Rz1*] (N=12). Plants were either inoculated with a non-resistance-breaking (BNYVV-ALHG) or with a resistance-breaking (BNYVV-AYPR) clone. Lateral roots were harvested 6 weeks after inoculation. The numbers below each bar indicate the infection rate (infected/inoculated). Mean values were calculated using only infected plants, whereas all values were averaged when no plant was infected. Error bars indicate standard deviation. The healthy controls are not shown because the absorbance values were zero. Significant differences between both tetrads were determined by the Wilcoxon test and are indicated by small letters (p < 0.05).

Next, we validated our aforementioned results with *Rz1* genotypes from different breeding companies with different genetic background. Previous resistance tests (unpublished data) using natural soils were indicative for genotypic variability with some *Rz1* genotypes sustaining some detectable virus, as shown by ELISA, although displaying complete tolerance to yield loss in the field. For this purpose, a susceptible and four *Rz1* genotypes were inoculated with the non-resistance-breaking (BNYVV-ALHG) and the resistance-breaking (BNYVV-AYPR) clone (**Figure 2**). Both clones produced similar ELISA values in the susceptible genotype A. In contrast, no virus accumulation was detectable in the two resistant genotypes B and D with the clone BNYVV-ALHG, whereas the resistance-breaking clone (BNYVV-AYPR) could overcome *Rz1*, which was also confirmed by statistical analysis. Surprisingly, we could determine in the resistant genotypes C and E a very low infection rate and ELISA values even after inoculation of BNYVV-ALHG. However, the ELISA values markedly increased after inoculation of BNYVV-AYPR demonstrating the resistance-breaking effect. These results were supported by statistical significance, which shows that agrobacterium-mediated infection of BNYVV and resistance-breaking work independent of the *Rz1* genotype.

### Screening of P25 tetrads for *Rz1* resistance-breaking properties

All tetrads analyzed in this study have been previously found in natural A, B and P type populations of BNYVV, but their resistance-breaking capacity has not been confirmed yet. In total, the resistance-breaking ability of 25 tetrads was tested in a susceptible and *Rz1* resistant genotype. We included AYPR, VCHG and VLHG as control, because their resistance-breaking properties have been shown before by reverse genetics with mechanical inoculation of recombinant viruses (Liebe et al., 2020) but not by direct agroinoculation as applied here. The large number of tetrads required several separated experiments, and therefore, a statistical comparison was performed between both genotypes inoculated with the same tetrad. Moreover, comparing ELISA values from plants infected with different tetrads was not performed, as the samples were not analyzed together. After each experiment, we confirmed the presence of the tetrads in the susceptible and resistant genotype by means of conventional sanger sequencing. Interestingly, seven tetrads were unstable in sugar beet (**Table 1**). The mutants were relatively quickly outcompeted by spontaneous occurring mutations during viral replication as indicated by multiple peaks in the electropherogram. The susceptible genotype was still infected, but some of the unstable tetrads were associated with a decreased pathogenicity indicated by a low infection rate (AFHG, AHHG, AYHG, SYHG) or a low ELISA values (AYHG). The second group comprised stable tetrads with no resistance-breaking properties due to the absence of virus detection by ELISA in the *Rz1* genotype. Similar to the aforementioned group, some tetrads were associated with a lower infection or a low mean ELISA value (AYHR, AYPG, AYRV). In contrast, the tetrads ACHR, AHHR and ASHR did not affect the pathogenicity of the A type clone. The last group contained only tetrads that were stable and allowed an accumulation in the *Rz1* genotype. A direct statistical comparison showed that ELISA values were for some tetrads not different between both genotypes, which indicates a similar accumulation level (e.g. AYPR, -DHG/D-HG, TFPR, TYPR, VHPG). Interestingly, even a deletion of one amino acid in the tetrad (-DHG/D-HG) allowed BNYVV to overcome *Rz1*. The other tetrads (AFPR, TCHG, VCHG, VHHG, VLHG) also mediated resistance-breaking, but the ELISA values in *Rz1* plants were significantly lower compared to the susceptible genotype. This was most apparent for TCHG with a mean ELISA absorbance of 0.74 in the susceptible genotype and 0.279 in the *Rz1* genotype. Finally, evaluation of leaf symptoms in the susceptible genotype revealed no effect of the tetrad (**Supplementary Figure S1**), but in some cases we could not observe leaf symptoms indicative for systemic movement (i.e. AYHR, SYHG).

**Table 1:** Effect of different P25 tetrads (aa67-70) on BNYVV accumulation of in a susceptible and *Rz1* resistant genotype.

	Susceptible genotype			*Rz1* resistant genotype			
Tetrads^a^	Infected/inoculated plants	Mean_405nm_ ^b^	SD^c^	Infected/inoculated plants	Mean_405nm_ ^b^	SD^c^	p value^d^
*Unstable tetrads*							
ACHG	10/18	0,584	0,31	0/18	–	–	–
AFHR	10/18	0,408	0,25	1/18	0,105	–	–
AFHG	6/18	0,752	0,42	0/18	–	–	–
AHHG	4/18	0,412	0,08	0/18	–	–	–
AYHG	4/18	0,216	0,071	0/18	–	–	–
SYHG	5/18	0,624	0,421	2/18	0,145	0,034	–
VYHG	15/18	0,524	0,288	4/18	0,199	0,107	–
*No accumulation in Rz1*							
ACHR	17/18	0,68	0,292	0/18	–	–	–
AHHR	12/18	0,771	0,273	0/18	–	–	–
ASHR	17/18	1,077	0,393	0/18	–	–	–
AYHR	4/18	0,255	0,055	0/18	–	–	–
AYPG	6/18	0,532	0,281	0/18	–	–	–
AYRV	11/18	0,374	0,274	0/18	–	–	–
*Accumulation in Rz1*							
AFPR	13/18	0,388	0,131	10/18	0,257	0,085	0.025*
AYPR	18/18	0,662	0,081	17/18	0,617	0,16	0.428
-DHG/D-HG	13/18	0,75	0,272	8/18	0,725	0,173	0.690
TCHG	15/18	0,74	0,162	6/18	0,279	0,252	0.001*
TFPR	17/18	0,678	0,216	17/18	0,669	0,221	0.917
TYHR	3/18	0,321	0,102	2/18	0,364	0,319	1
TYPR	18/18	0,597	0,196	17/18	0,576	0,158	0.503
VCHG	18/18	0,652	0,1	13/18	0,473	0,146	0.001*
VFHG	16/18	1,143	0,33	12/18	0,524	0,26	0.001*
VHHG	17/18	0,662	0,103	15/18	0,427	0,208	0.001*
VHPG	18/18	0,701	0,203	17/18	0,75	0,11	0.541
VLHG	18/18	0,64	0,091	9/18	0,409	0,175	0.001*

a The presence of all tetrad variants was confirmed by sequencing of P25 isolated from a susceptible and a resistant plant at the end of the incubation period (5 – 6 weeks). Unstable tetrad variants were identified by multiple peaks in the electropherogram at this position.

b The virus accumulation was determined by ELISA. Mean values were calculated including only infected plants.

c SD= standard deviation

d The p value indicates significant differences between both genotypes using the Wilcoxon test. Only tetrad variants with resistance-breaking properties were included into the analysis. Significant differences (p < 0.05) are indicated by “*”, -: not applicable.

### Role of second site mutations for functionality of P25 tetrads

**Figure 3:**
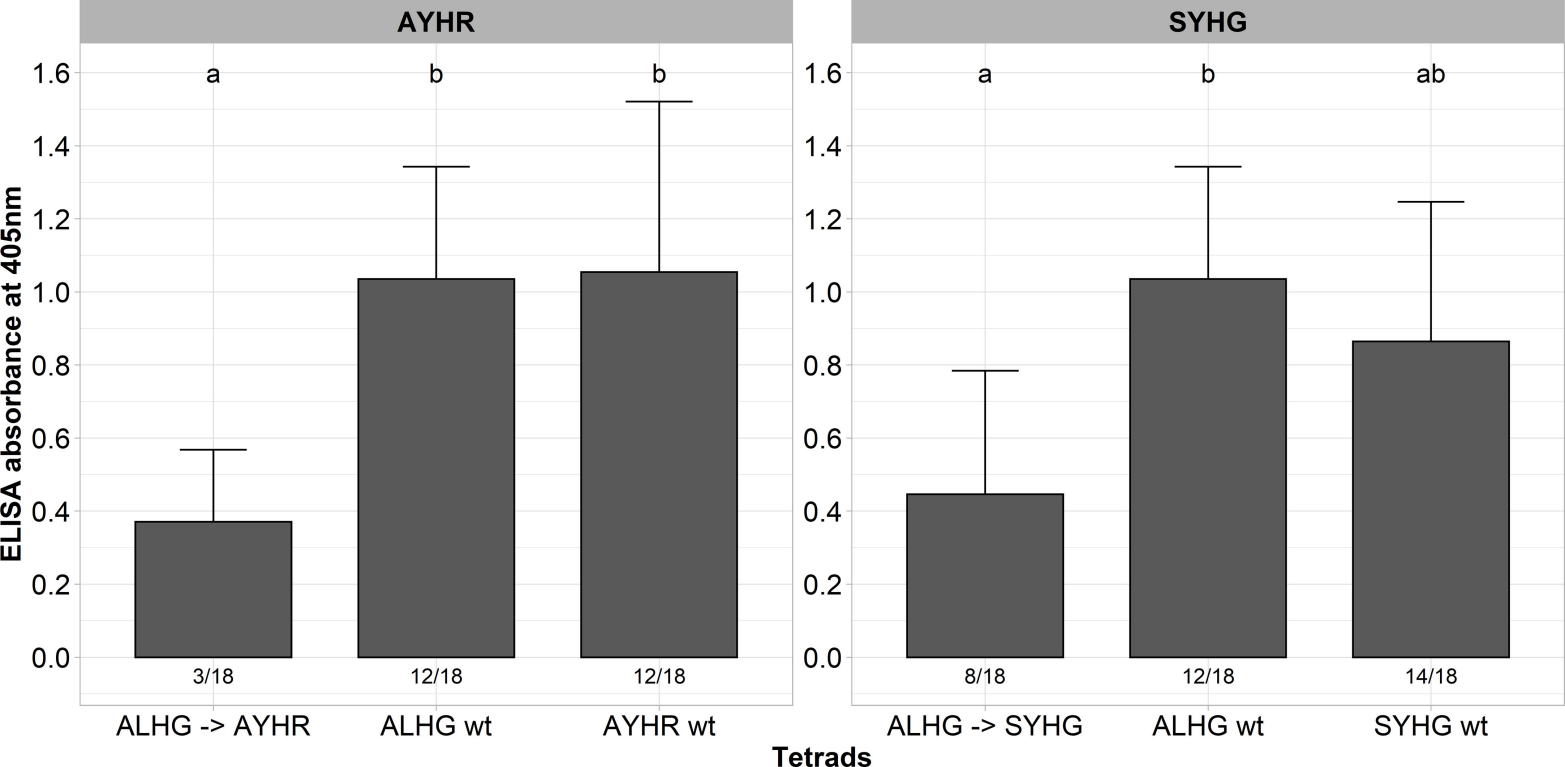
ELISA absorbance values for BNYVV measured in lateral roots of plants from a susceptible sugar beet genotype (N=18). Plants were inoculated with the wild type clone (ALHG wt) and with different BNYVV variants in which either the tetrad ALHG of the A type P25 was mutated (ALHG → AYHG/SYHG) or the whole ORF from A type P25 was replaced by an ORF encoding a P25 from a B (AYHR wt) or P type (SYHG wt). Lateral roots were harvested 6 weeks after inoculation. The numbers below each bar indicate the infection rate (infected/inoculated). Mean values were calculated using only infected plants, whereas all values were averaged when no plant was infected. Error bars indicate standard deviation. The healthy controls are not shown because the absorbance values were zero. The statistical analysis was done separately for AYHR (left) and SYHG (right) with the Dunnett *post hoc* test. Significant differences are indicated by small letters (p < 0.05).

A described above, some tetrads impaired the functionality of P25 as indicated by instability of the introduced mutations, low infection rate and low ELISA values. In general, P25 sequences from A, B and P type populations display many different mutations besides the hypervariable tetrad, but we did not consider their effect on the functionality of P25 in the above-described experiments. A multiple alignment of the P25 sequence from our A type clone (ALHG) with wild type sequences from the P (SYHG) and B (AYHR) type show many mutations outside the tetrad (**Supplementary Figure 2**). Interestingly, we observed in the above-described experiments that the pathogenicity of our A type clone was either reduced (AYHR) or the tetrad displayed instability (SYHG). Therefore, we speculated that these site mutations outside the tetrad are critical for the function of P25. To evaluate this hypothesis, we cloned the entire ORF from the P and B type P25 into the background of the A type RNA3 non-coding sequence. Both mutated RNA3s were tested for infectivity in the susceptible genotype along with the A type clone carrying either ALHG, AYHR and SYHG as tetrad. As observed before, the replacement of the tetrad ALHG by AYHR or SYHG in the A type P25 was associated with a drop of ELISA values compared to the ALHG wt (**Figure 3**). In contrast, replacing the entire ORF from the A type P25 by a B or P type P25 did not compromise the pathogenicity. Moreover, both B and P type P25 displayed a similar infection rate and ELISA values as the P25 ALHG wt.

### Effect of different RNA5 species on *Rz1* resistance-breaking

**Figure 4:**
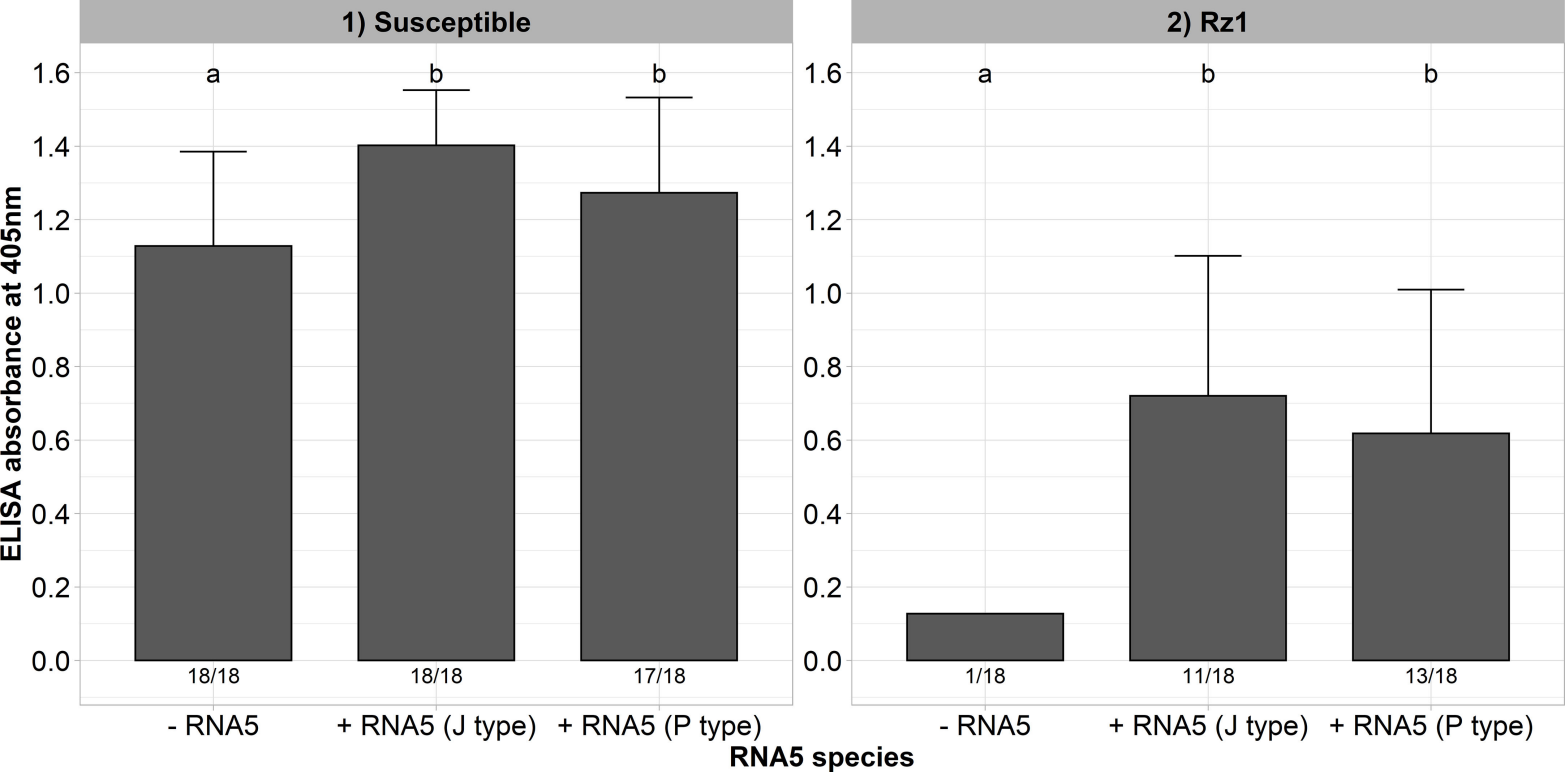
ELISA absorbance values for BNYVV measured in lateral roots of plants from a susceptible and *Rz1* resistant sugar beet genotype (N=18). The non-resistance-breaking clone with the tetrad ALHG was either inoculated alone (-RNA5) or together with the P or J type RNA5 (+ RNA5). Lateral roots were harvested 6 weeks after infection. The numbers below each bar indicate the infection rate (infected/inoculated). Mean values were calculated using only infected plants, whereas all values were averaged when no plant was infected. Error bars indicate standard deviation. The healthy controls are not shown because the absorbance values were zero. The statistical analysis between tetrad variants was done separately for both genotypes with the Dunnett *post hoc* test. Significant differences are indicated by small letters (p < 0.05).

In a previous study, we could identify natural A and B type populations in Europe harboring an RNA5 belonging either to the J or P type (Liebe and Varrelmann, 2022). It was supposed that this genetic reassortment allows BNYVV to overcome *Rz1* independent of the tetrad. Both RNA5 species display a high sequence similarity in the P26 ORF suggesting a similar effect on *Rz1* stability (**Supplementary Figure 3**). To provide experimental evidence for our hypothesis, we cloned both RNA5 species from populations with resistance-breaking properties. A susceptible and *Rz1* resistant genotype was inoculated with and without the RNA5s (**Figure 4**). The presence of both RNA5s increased the virus accumulation in the susceptible genotype significantly as indicated by the ELISA values. Except for one plant, the A type clone was not able to establish an infection in plants from the resistant genotype when the RNA5 was absent from the inoculum. In contrast, the addition of either P or J type RNA5 allowed BNYVV to establish an infection in many plants of the resistant genotype. However, it is noticeable that these plants displayed ELISA values that were still lower than in the susceptible genotype. Similarly, the infection rate was clearly reduced in the resistant genotype.

**Figure 5:**
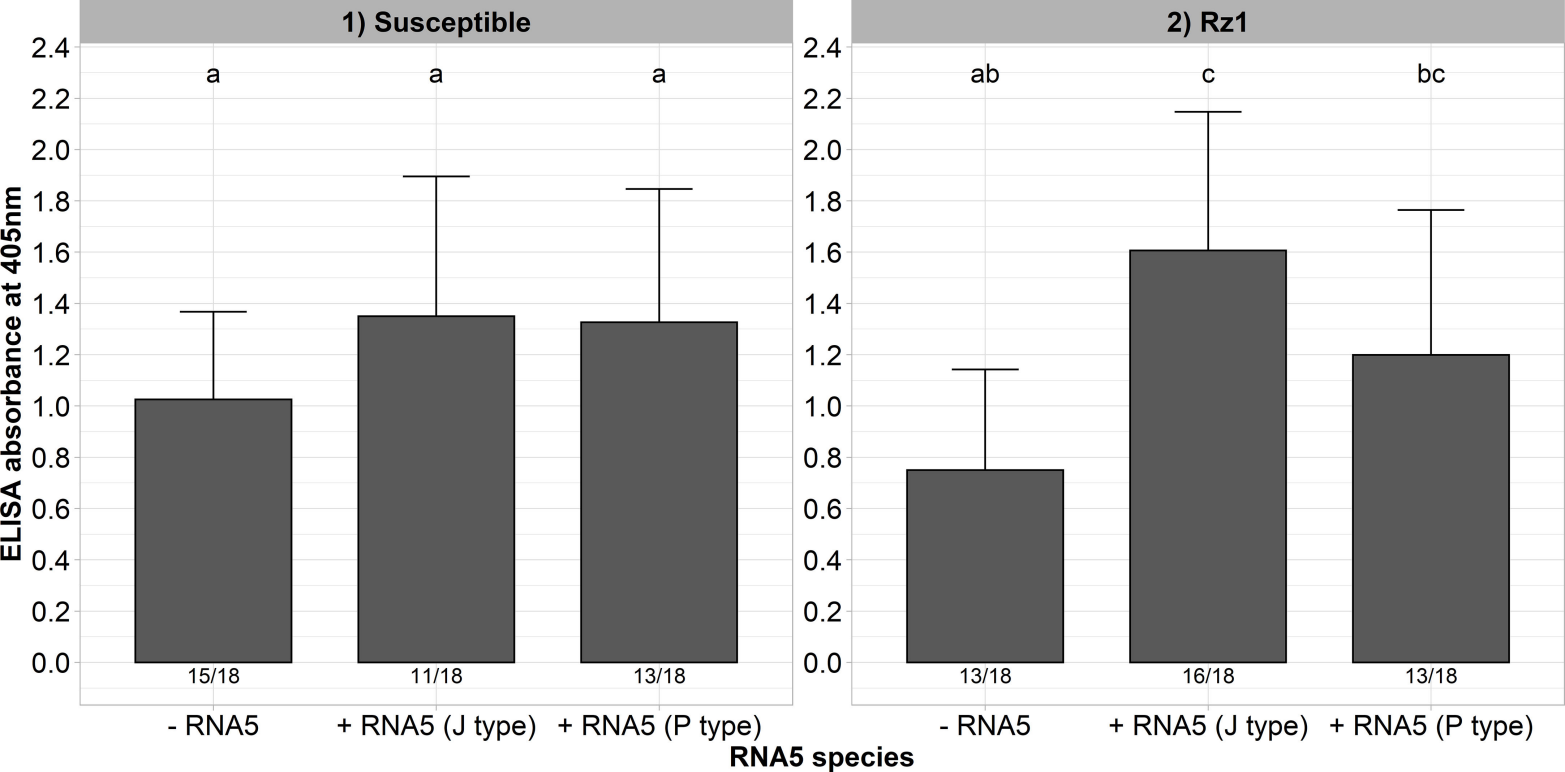
ELISA absorbance values for BNYVV measured in lateral roots of plants from a susceptible and *Rz1* resistant sugar beet genotype (N=18). The resistance-breaking clone with the tetrad AYPR was either inoculated alone (-RNA5) or together with the P or J type RNA5 (+ RNA5). Lateral roots were harvested 6 weeks after infection. The numbers below each bar indicate the infection rate (infected/inoculated). Mean values were calculated using only infected plants, whereas all values were averaged when no plant was infected. Error bars indicate standard deviation. The healthy controls are not shown because the absorbance values were zero. The statistical analysis was done separately for both genotypes with the Dunnett *post hoc* test. Significant differences are indicated by small letters (p < 0.05).

The aforementioned results showed that our A type clone supplemented with an RNA5 is able to overcome *Rz1* independent of a mutation in the tetrad which suggests different resistance-breaking mechanism. Interestingly, at least in the susceptible genotype there was an additional effect of the RNA5 on the measured ELISA values. To provide further evidence for this effect, we inoculated the resistance-breaking clone (AYPR) together with both RNA5 types into the susceptible and *Rz1* genotype. This clone is not limited in its replication in the *Rz1* genotype, and therefore, should resemble this effect as observed before in the susceptible genotype. Here again, the ELISA values increased in both genotypes when either the RNA5 from J or P type was supplemented to the inoculum (**Figure 5**). This effect was most pronounced in the *Rz1* genotype. However, the effect was only significant in case of the J type RNA5 in the *Rz1* genotype.

### *Rz2* effectively controls *Rz1* resistance-breaking variants

**Figure 6:**
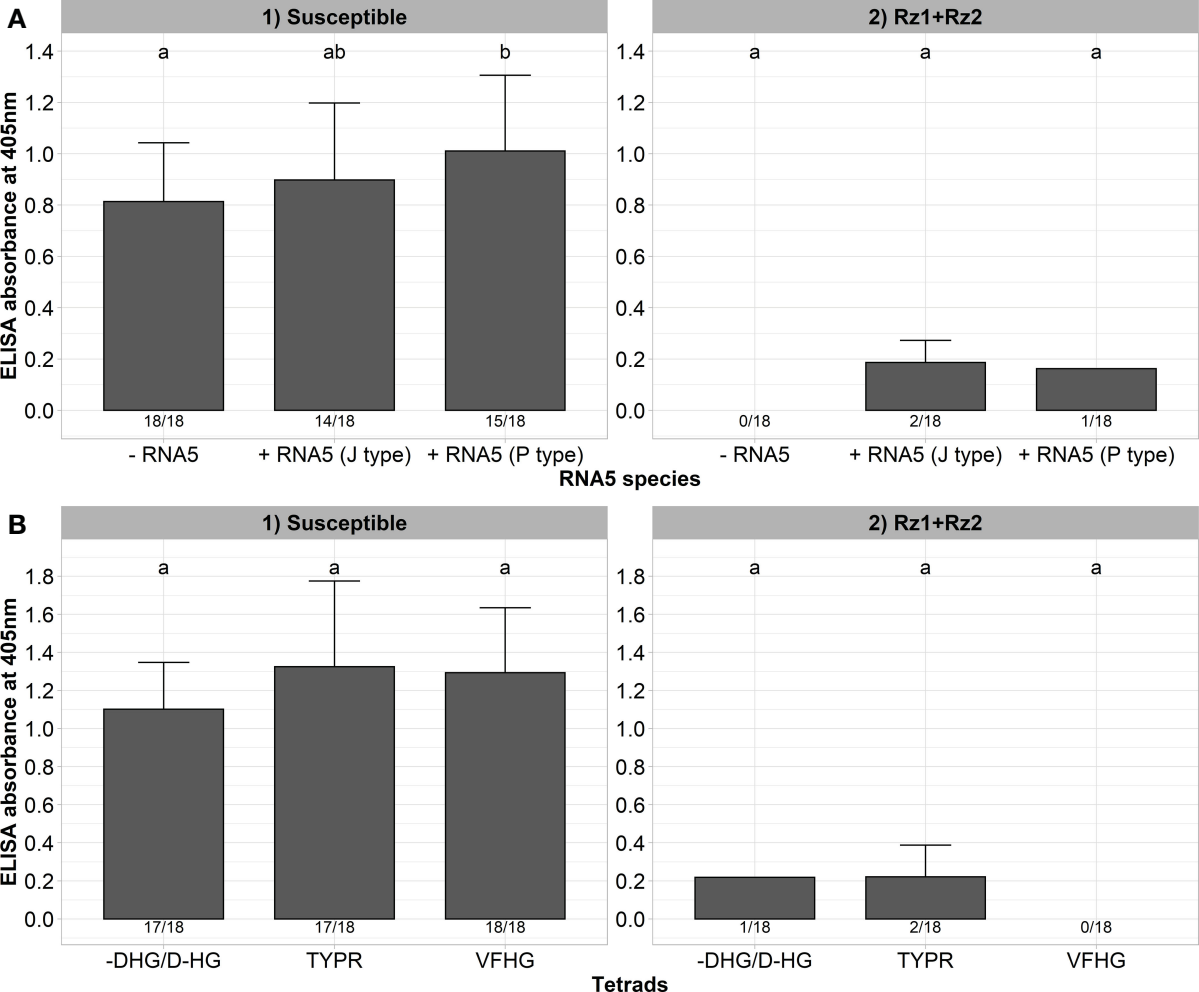
**(A)** ELISA absorbance values for BNYVV measured in lateral roots of plants from a susceptible and *Rz1*+*Rz2* resistant sugar beet genotype (N=18). The non-resistance-breaking clone with the tetrad ALHG was either inoculated alone (-RNA5) or together with the P or J type RNA5 (+ RNA5). Lateral roots were harvested 6 weeks after inoculation. **(B)** ELISA absorbance values for BNYVV measured in lateral roots of plants from a susceptible and *Rz1*+*Rz2* resistant sugar beet genotypes (N=18). Resistance-breaking clones with different tetrads (VFHG, -DHG/D-HG, TYPR) were inoculated into both genotypes. Lateral roots were harvested 6 weeks after infection. The numbers below each bar indicate the infection rate (infected/inoculated). Mean values were calculated using only infected plants, whereas all values were averaged when no plant was infected. Error bars indicate standard deviation. The healthy controls are not shown because the absorbance values were zero. The statistical analysis was done separately for both genotypes with the Dunnett *post hoc* test. Significant differences are indicated by small letters (p < 0.05).

Finally, we wanted to determine whether the resistance-breaking effect of RNA5 and P25 mutations of the tetrad are specific to *Rz1* and have no effect on virus accumulation in double resistant *Rz1* and *Rz2* sugar beet genotypes. For this purpose, we inoculated our A type clone together with the J or P type RNA5 into a susceptible and resistant genotype harboring *Rz1* and *Rz2* (**Figure 6A**). As previously observed, the presence of RNA5 increased the ELISA values measured in the susceptible genotype, but this effect was not consistent for both RNA5 species. Furthermore, the presence of either J or P type RNA5 in the inoculum had no effect on the infection of the double resistant genotype. Only 1 (P type) and 2 (J type) plants were infected, but the ELISA values were very low (0,125 - 0,247). In the next step, we inoculated clones with different *Rz1* resistance-breaking tetrads (VFHG, -DHG/D-HG, TYPR) into the double resistant genotype to prove their effective control by *Rz2*. As observed before, all clones were able to induce a strong infection in the susceptible genotype (**Figure 6B**). In contrast, the infection rate and the ELISA values dropped dramatically after inoculation into the double resistant genotype. Only the clones with the tetrads TYPR and -DHG/D-HG were able to establish an infection in a few plants, but the ELISA values were very low (0,103 – 0,338) compared to the susceptible genotype. Furthermore, the phenotype of inoculated plants from the double resistant genotype was comparable with the healthy control whereas susceptible plants showed clear rhizomania symptoms (**Supplementary Figure 4**).

## Discussion

In this study, we combined a reverse genetic system for BNYVV with direct agroinoculation of sugar beet seedlings to perform a comprehensive analysis of the P25 amino acids involved in *Rz1* resistance-breaking. It has to be pointed out that the virus titer was not assessed in this study, as no standard for virus quantification was available. Therefore, any comparisons of ELISA values between individual experiments were not done. Differences between ELISA values from treatments of the same experiment are only indicative for the relative virus titer. Despite this limitation, the results presented in this study allow important conclusions on resistance-breaking by BNYVV.

First, we confirmed that the BNYVV A type clone derived from a non-resistance-breaking population is indeed not able accumulate in *Rz1* resistant plants after direct agroinoculation which allows a clear discrimination from the susceptible genotype. Subsequently, the successful transformation of the non-resistance breaking clone of BNYVV into an *Rz1* resistance-breaking clone was demonstrated by replacing the tetrad ALHG with AYPR. Furthermore, the resistance-breaking ability was tested with several *Rz1* genotypes derived from different breeding companies. Although the gene encoding *Rz1* is not known, it is believed that all breeders used the same source for introducing *Rz1* into their varieties. This is supported by our results as the resistance-breaking clone with the tetrad AYPR was able to accumulate in all *Rz1* genotypes to a level comparable to the susceptible genotype. Interestingly, we noticed that the non-resistance-breaking clone with the tetrad ALHG was able to accumulate in several plants of two *Rz1* genotypes, whereas other genotypes were not infected at all. The ELISA values were still very low and increased dramatically after replacing the tetrad ALHG with AYPR. However, this indicates that the genetic background of the genotype somehow affects the ability of *Rz1* to prevent an infection with the non-resistance-breaking clone of BNYVV. It remains to be shown whether some minor genes or allelic variation of *Rz1* is responsible for this reaction.

After validation of our infection assay, we performed a comprehensive screening for *Rz1* resistance-breaking tetrads which revealed multiple new variants allowing BNYVV to overcome *Rz1*. Among them, -DHG/D-HG, TFPR, TYPR, VFHG, VHHG and VHPG displayed clear *Rz1* resistance-breaking properties as indicated by positive ELISA values and high infection rates in *Rz1* plants. This highlighted the suitability of our assay to test the resistance-breaking effect of newly emerging tetrads. For example, the tetrad -DHG/D-HG with a single amino acid deletion was found in resistance-breaking A-type populations from Turkey (Yilmaz et al., 2018) and we could indeed confirm their effect on *Rz1*. For some tetrads (AFPR, TCHG and TYHR) it was not possible to deduce a clear resistance-breaking property as plants of the resistant genotype were infected, but the ELISA values were very low. However, only a quantitative DAS-ELISA with a virus standard would allow a definite conclusion, as already discussed above. Apart from that, a great portion of the tested tetrads displayed no resistance-breaking properties at all. However, some tetrads were either unstable in the background of our A type clone or decreased dramatically the virus pathogenicity. This was most apparent for the tetrad AYHR that is exclusively found in B type isolates of BNYVV (Liebe and Varrelmann, 2022). As P25 sequences from A, B and P type isolates differ in many amino acid positions (Liebe and Varrelmann, 2022), we hypothesized that these mutations are critical to retain the function of P25. Indeed, we were able to show that the wild type P25 ORF from the B (AYHR) and P (SYHG) is functional in the A type background. Moreover, the ELISA values were comparable to the wild type A type clone without any reduction in pathogenicity as observed when the tetrad ALHG was just replaced by AYHR or SYHG. This suggested that each virus type of BNYVV with its specific P25 sequence is limited in its ability to mutate the tetrad as it can impair the function of P25. This is supported by a previous study which has shown that an exchange of the tetrad in the B-type P25 reduced symptom development, P25 accumulation and self-oligomerization in the experimental host *Tetragonia expansa* (Klein et al., 2007). Based on our results, it seems that the P25 of the BNYVV A type is able to tolerate the replacement of many different tetrads. This would also be an explanation for the large tetrad variability observed in A type populations rather than in B type populations (Schirmer et al., 2005). However, our recent results from analysis of natural populations show that BNYVV can form genetic reassortments allowing the exchange of the complete RNA3 between different virus types (Liebe and Varrelmann, 2022). Whether this allows a B type population to overcome *Rz1* with a resistance-breaking RNA3 from the A type needs to be shown with a reverse genetic system, but this is currently not possible as no cDNA clone of the BNYVV B type is available.

We have shown in a previous study that natural *Rz1* resistance-breaking A and B type populations can harbor an additional RNA5 belonging either to the P or J type group (Liebe and Varrelmann, 2022). The P type RNA5 belongs originally to P type isolates and is the major driver of *Rz1* resistance-breaking by this virus type (Müllender et al., 2022). In contrast, evidence for the resistance-breaking effect of the RNA5 belonging to the J type group is based on experiments using natural virus populations (Nakagami et al., 2021; Tamada et al., 2021). Both RNA5 species display some sequence variation in the encoded P26 protein, and therefore, we wanted to compare the resistance-breaking properties of both in the background of our BNYVV A type clone. Independently of the RNA5 species, BNYVV was able to overcome *Rz1*, but the ELISA values were still lower compared to the susceptible genotype. A similar observation has been also made when the resistance-breaking ability of the P type RNA5 was investigated with a cDNA clone of the BNYVV P type (Müllender et al., 2022). Moreover, natural populations with an RNA5 can be able to accumulate to the same extent in both genotypes (susceptible and resistant), whereas other populations display still a somewhat reduced virus content in resistant plants (Liebe and Varrelmann, 2022). The reason for this phenomenon is unknown, but it strongly suggests that further mutations are involved in resistance-breaking besides the presence of P26 encoding RNA5. Finally, our results also indicate a positive effect of the RNA5 on the virus pathogenicity. This effect was not consistently statistically significant, but it was observed for both RNA5 species and genotypes in independent trials. The RNA5 encoded protein P26 shares sequence homology with P25 supporting an additional role in virus pathogenicity. Our reverse genetic system enables us now to study the exact P26 function in further trials.

The worldwide occurrence of *Rz1* resistance-breaking populations constitutes a threat to sugar beet production. This strengthens the importance of the second resistance gene *Rz2* that is used in some cultivars in combination with *Rz1*. A previous study showed that *Rz1*+*Rz2* cultivars did not offer complete protection against BNYVV under field conditions (Galein et al., 2018), although there are no reports on *Rz2* resistance-breaking populations yet. To test this finding, we tested the ability of our *Rz1* resistance-breaking clones to accumulate in a double resistant genotype (*Rz1*+*Rz2*). We tested both resistance-breaking mechanisms separately (tetrad mutation and addition of RNA5), but none of the clones was able to initiate an infection in the double resistant comparable to the susceptible genotype. Consequently, the resistance-breaking mechanisms described here are highly specific to *Rz1*, and provide no fitness advantage to the virus in double resistant genotypes. This conclusion is further supported by a recent study showing that the triple gene block 1 protein from BNYVV is recognized by *Rz2* leading to a hypersensitive response (Wetzel et al., 2021). In contrast, it is believed that P25 is the avirulence gene of *Rz1*. The observation of a partial protection against BNYVV by double resistant cultivars (Galein et al., 2018) might be attributed to specific field conditions when other factors compromise the resistance like a secondary infection with e.g. nematodes or other pathogens.

**Figure 7:**
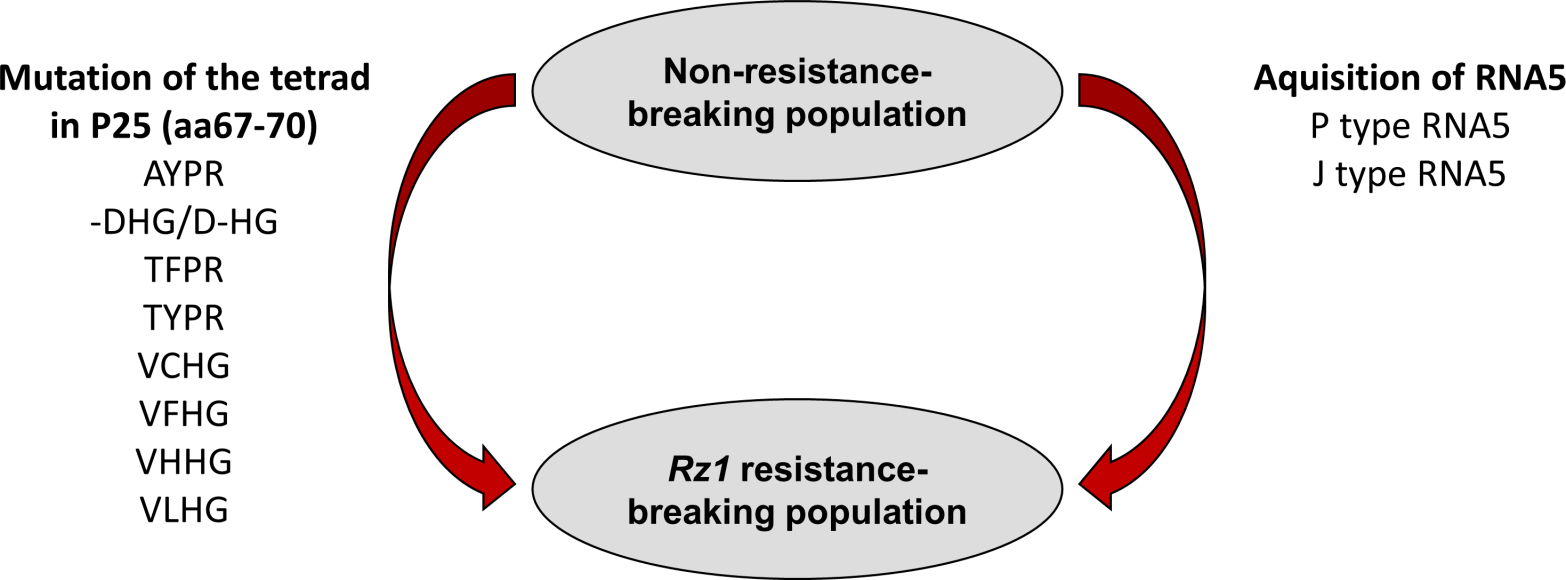
Scheme illustrating the resistance-breaking mechanisms utilized by BNYVV to overcome *Rz1* resistance.

In summary, based on previous studies using natural virus populations it has been proposed that *Rz1* resistance-breaking occurs either by mutation of the tetrad or by introduction of an additional RNA5 species into the virus population. We were able to confirm both mechanisms with our reverse genetic system leading to a model summarizing how a BNYVV A type population can overcome *Rz1* (**Figure 7**). It highlights the flexibility of the viral genome which is the driver of the arms race between BNYVV and host resistance in sugar beet. Consequently, it also strengthens the need for continuous research on the identification of new resistance genes. The BNYVVA type clone in combination with direct agroinoculation of young sugar beet seedlings is a valuable tool for breeder to perform to such comprehensive analysis.

## Data availability statement

The original contributions presented in the study are included in the article/**Supplementary Material**. Further inquiries can be directed to the corresponding author.

## Author contributions

SL and MV planned the experiments. SL conducted the experiments and was responsible for data analysis. EM created RNA3 clones for the tetrad screening. SL wrote the manuscript with the collaboration of all authors. All authors contributed to the article and approved the submitted version.
