# Supplementary material for: The arms race between beet necrotic yellow vein virus and host resistance in sugar beet
Source: Front Plant Sci. 2023 Mar 31;14:1098786. doi: 10.3389/fpls.2023.1098786 (PMC10102433; doi:10.3389/fpls.2023.1098786)
Supplement: Supplementary file 1 [file DataSheet_1.docx]

Supplementary Material

Supplementary Table 1. Primers used to replace the tetrad ALHG by other variants in the P25.

| **Tetrad** | **Name** | **Sequence forward primer (5’-3’)** | **Name** | **Sequence reverse primer (5’-3’)** |
| --- | --- | --- | --- | --- |
| AYHR | AYHRfor | GTGTGCTTATCATCGTCCTTATTGTGGGTTTCGTGCC | AYHRrev | AAGGACGATGATAAGCACACAATAATCCACGAAACC |
| SYHG | SYHGfor | GTGTTCTTATCATGGGCCTTATTGTGGGTTTCG | SYHGrev | AAGGCCCATGATAAGAACACAATAATCCACGAAACC |
| AFPR | AFPRfor | ATTGTGTGCTTTTCCTCGTCCTTATTGTGGGTTTCGTGC | AFPRrev | AAGGACGAGGAAAAGCACACAATAATCCACGAAACC |
| VYHG | VYHGfor | GTGTGTTTATCATGGGCCTTATTGTGGGTTTCGTGC | VYHGrev | AAGGCCCATGATAAACACACAATAATCCACGAAACC |
| VHHG | VHHGfor | GTGTGTTCATCATGGGCCTTATTGTGGGTTTCGTGC | VHHGrev | AAGGCCCATGATGAACACACAATAATCCACGAAACC |
| -DHG/D-HG | DHGfor | TTGTGTGATCATGGGCCTTATTGTGGGTTTCGTGC | DHGrev | AATAAGGCCCATGATCACACAATAATCCACGAAACC |
| TLHG | A_TLHGfor | GTGTACTCTTCATGGGCCTTATTGTGGGTTTCGTGC | A_TLHGrev | AGGCCCATGAAGAGTACACAATAATCCACGAAACC |
| AFHR | A_AFHRfor | GTGTGCTTTTCATAGGCCTTATTGTGGGTTTCGTGC | A_AFHRrev | AGGCCTATGAAAAGCACACAATAATCCACGAAACC |
| VYHR | A_VYHRfor | GTGTGTTTATCATAGGCCTTATTGTGGGTTTCGTGC | A_VYHRrev | AGGCCTATGATAAACACACAATAATCCACGAAACC |
| AHHG | A_AHHGfor | GTGTGCTCATCATGGGCCTTATTGTGGGTTTCGTGC | A_AHHGrev | AGGCCCATGATGAGCACACAATAATCCACGAAACC |
| TFPR | A_TFPRfor | GTGTACTTTTCCTAGGCCTTATTGTGGGTTTCGTGC | A_TFPRrev | AGGCCTAGGAAAAGTACACAATAATCCACGAAACC |
| AHHG | AHHGfor | GTGCTCATCATGGGCCTTATTGTGGGTTTCGTGC | AHHGrev | AAGGCCCATGATGAGCACACAATAATCCACGAAACC |
| ACHG | ACHGfor | GTGCTTGTCATGGGCCTTATTGTGGGTTTCGTGC | ACHGrev | AAGGCCCATGACAAGCACACAATAATCCACGAAACC |
| VHPG | VHPGfor | GTGTTCATCCTGGGCCTTATTGTGGGTTTCGTGC | VHPGrev | AAGGCCCAGGATGAACACACAATAATCCACGAAACC |
| TYPR | TYPRfor | GTACTTATCCTAGGCCTTATTGTGGGTTTCGTGC | TYPRrev | AAGGCCTAGGATAAGTACACAATAATCCACGAAACC |
| AFHG | AFGHfor | GTGCTTTTCATGGGCCTTATTGTGGGTTTCGTGC | AFGHrev | AAGGCCCATGAAAAGCACACAATAATCCACGAAACC |
| TCHG | TCHGfor | GTACATGTCATGGGCCTTATTGTGGGTTTCGTGC | TCHGrev | AAGGCCCATGACATGTACACAATAATCCACGAAACC |
| ACHR | ACHRfor | GTGCTTGTCATCGTCCTTATTGTGGGTTTCGTGC | ACHRrev | AAGGACGATGACAAGCACACAATAATCCACGAAACC |
| AYHG | AYHGfor | GTGCTTATCATGGGCCTTATTGTGGGTTTCGTGC | AYHGrev | AAGGCCCATGATAAGCACACAATAATCCACGAAACC |
| AYRV | AYRVfor | GTGCTTATCGTGTTCCTTATTGTGGGTTTCGTGC | AYRVrev | AAGGAACACGATAAGCACACAATAATCCACGAAACC |
| AHHR | AHHRfor | GTGCTCATCATCGTCCTTATTGTGGGTTTCGTGC | AHHRrev | AAGGACGATGATGAGCACACAATAATCCACGAAACC |
| AYPG | AYPGfor | GTGCTTATCCTGGGCCTTATTGTGGGTTTCGTGC | AYPGrev | AAGGCCCAGGATAAGCACACAATAATCCACGAAAC |
| TYHR | TYHRfor | GTACATATCATCGTCCTTATTGTGGGTTTCGTGC | TYHRrev | AAGGACGATGATATGTACACAATAATCCACGAAAC |
| ASHR | ASHRfor | GTGCTTCTCATCGTCCTTATTGTGGGTTTCGTGC | ASHRrev | AAGGACGATGAGAAGCACACAATAATCCACGAAACC |
|  | npEfor | CGGATTGTCCCTATACGAATTCATTAGACAGCCGCTTAGC | npErev | GCTAAGCGGCTGTCTAATGAATTCGTATAGGGACAATCCG |

Supplementary Table 2. Primers used to replace the P25 open reading frame (ORF) in the RNA3 of the A type clone.

| **Information** | **Forward primer** | **Sequence forward primer (5’-3’)** | **Reverse primer** | **Sequence reverse primer (5’-3’)** |
| --- | --- | --- | --- | --- |
| Amplification of the P25 ORF from B and P type. Bases in lower case letters are specific to the 5’ and 3’ end of the RNA. Overlapping bases to the vector are written in capital letters. | B-P25-fw | ATCATCATTAAGTGACCGTCatgggtgatatattaggcgcag | B-P25-rv | TTGAAATTGTGATAACTCTAatcatcatcatcaacaccgtcagg |
| Amplification and lineralization of the pDIVA plasmid. | BN-RNA3-OPEN-fw | TAGAGTTATCACAATTTCAACAACAC | BN-RNA3-OPEN-rv | GACGGTCACTTAATGATGATCAG |
| Amplification primers for the insert in pDIVA. Primers were also used for sequencing of the PCR product. | 274 | CAATCCCACTATCCTTCGCAAGACC | 314 | CCCTAATTCCCTTATCTGGGAACTAC |
| Internal primer for sequencing the BNYVV RNA3 in pDIVA. | P25_fw | ATGGGTGATATATTAGGCGCA |  |  |

Supplementary Table 3. Primers used to clone the RNA5 J and P type into the plasmid pDIVA.

| **Information** | **Forward primer** | **Sequence forward primer (5’-3’)** | **Reverse primer** | **Sequence reverse primer (5’-3’)** |
| --- | --- | --- | --- | --- |
| Amplification and lineralization of the pDIVA plasmid. | PAPA | CCTCTCCAAATGAAATGAACTTCCTTATATAG | MAMA | GGGTCGGCATGGCATCTCCACCTCCTC |
| Amplification of the full length RNA5 from J and P type. Bases in lower case letters are specific to the 5’ and 3’ end of the RNA. Overlapping bases to the vector are written in capital letters. | RNA5-fw | AGGAAGTTCATTTCATTTGGAGAGGaaattcaaagtactttcatattgtac | RNA5-rv | GAGATGCCATGCCGACCCttttttttttttttttttttttgtcaataca |
| Primer for sequencing of the insert in pDIVA. | 274 | CAATCCCACTATCCTTCGCAAGACC | 314 | CCCTAATTCCCTTATCTGGGAACTAC |

**
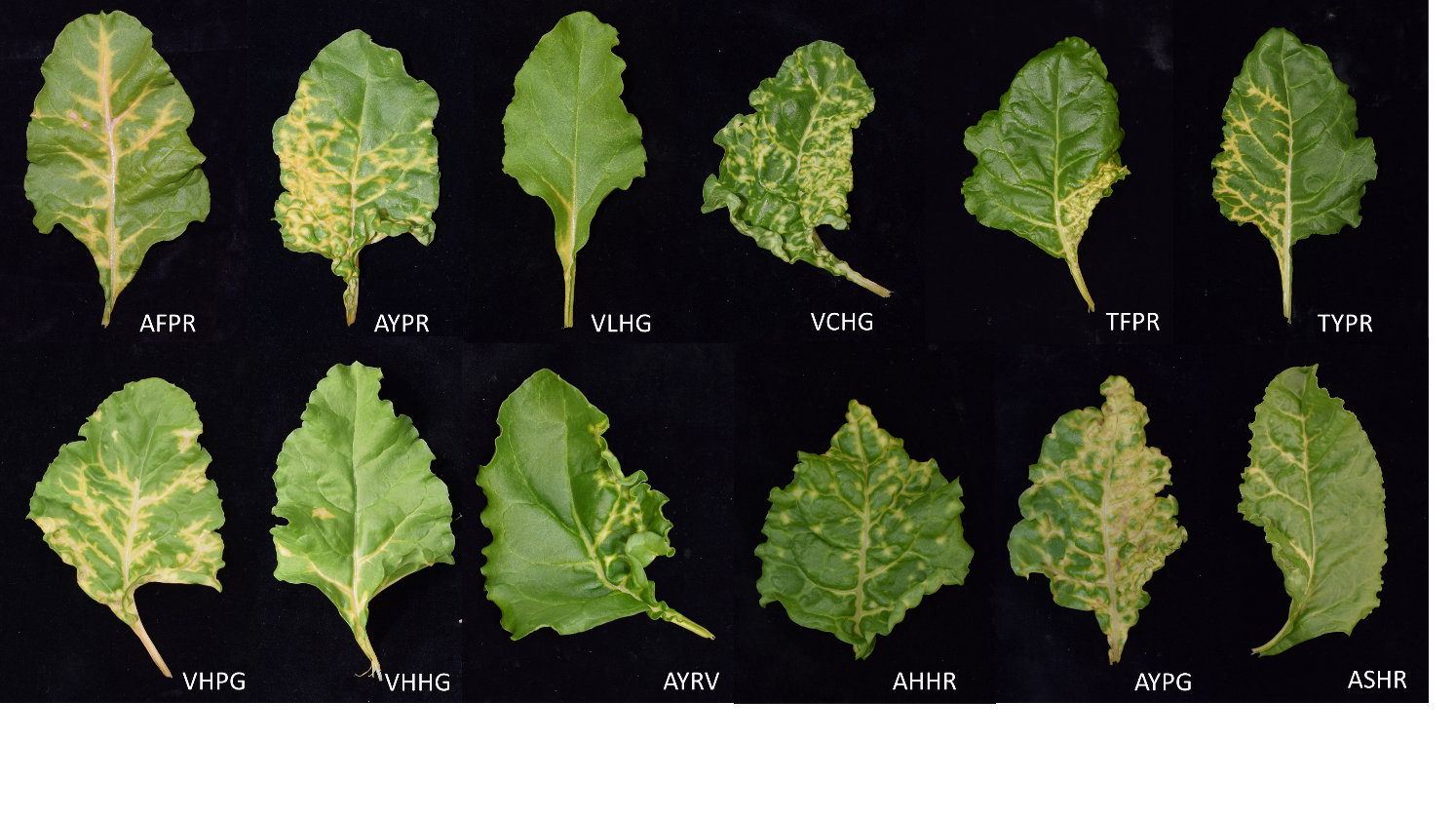
Supplementary Figure 1.** Systemic symptoms on sugar beet leaves from a susceptible genotype induced by the cDNA clone of BNYVV carrying different tetrad variants in the pathogenicity factor P25. Infected leaves showed vein yellowing and later also necrosis.


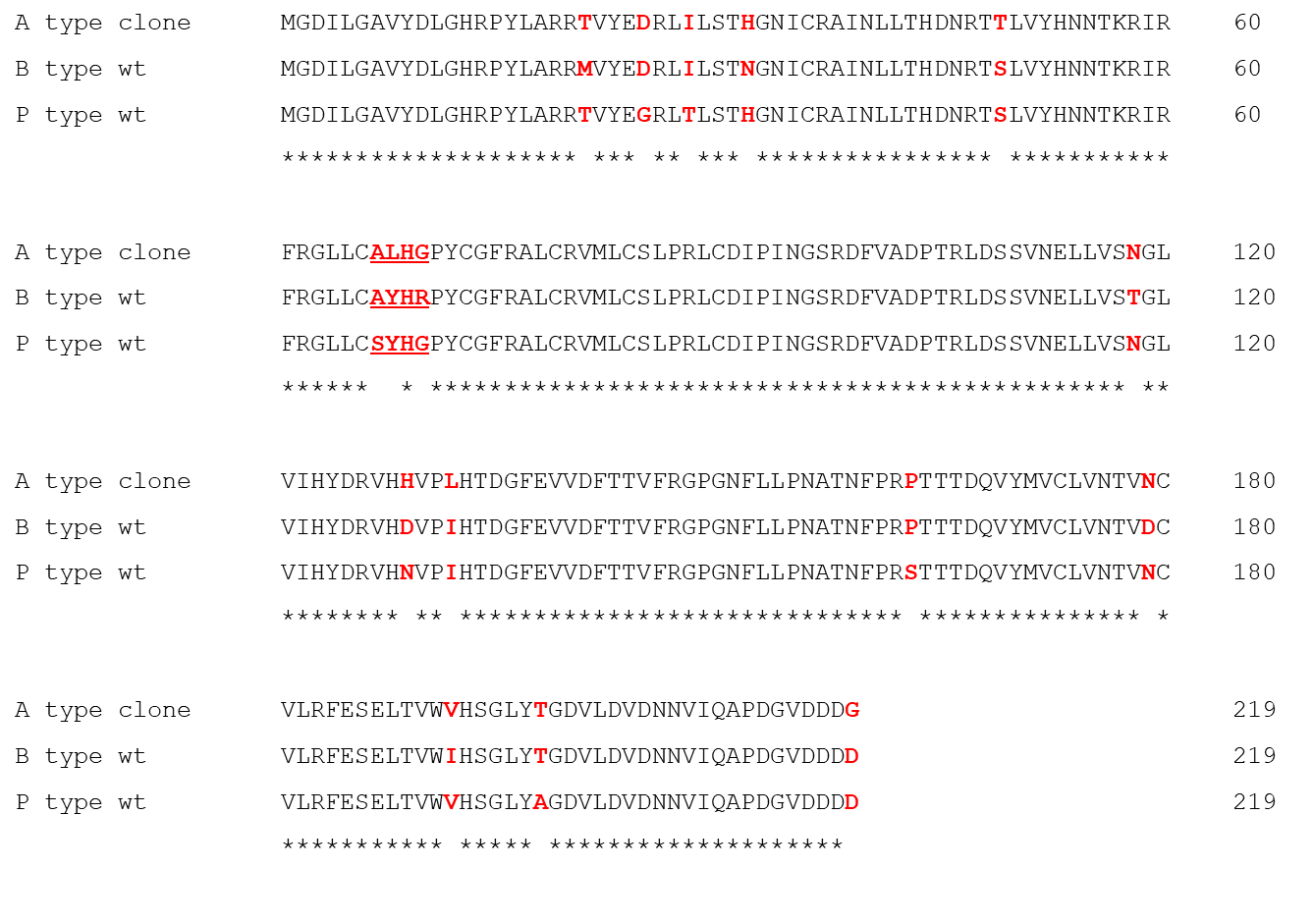


**Supplementary Figure 2.** Alignment of P25 amino acid sequences derived from the A type clone and wild type (wt) populations of the B and P type. Amino acid differences are highlighted in red and the tetrad sequence is additionally underlined.


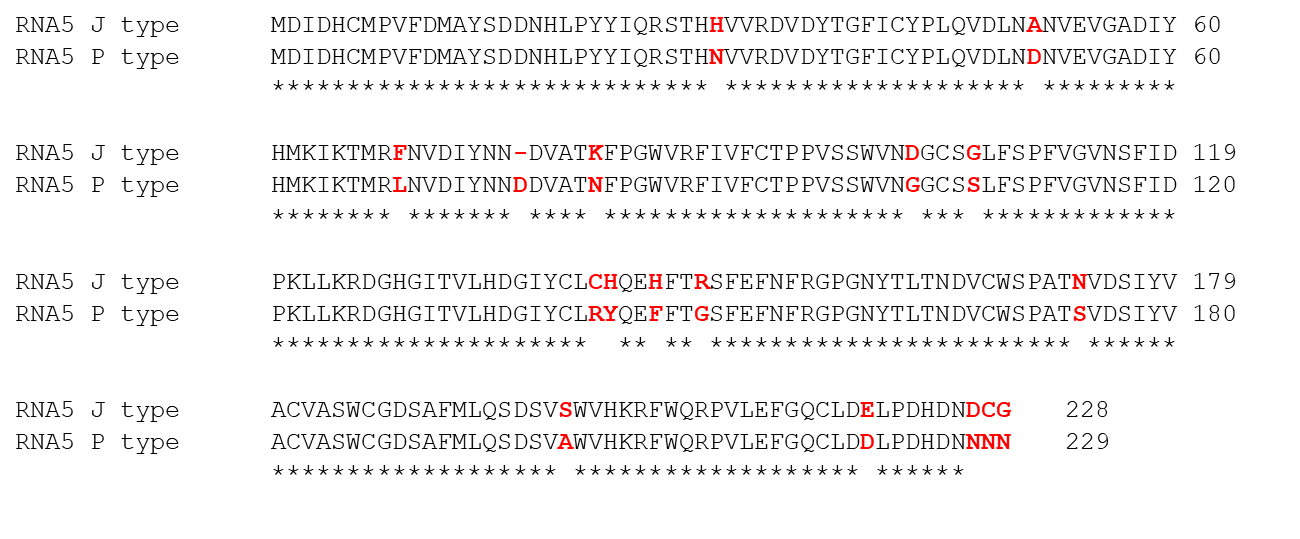
**Supplementary Figure 3.** Alignment of P26 amino acid sequences derived from BNYVV P and J type. Amino acid differences are highlighted in red.

**
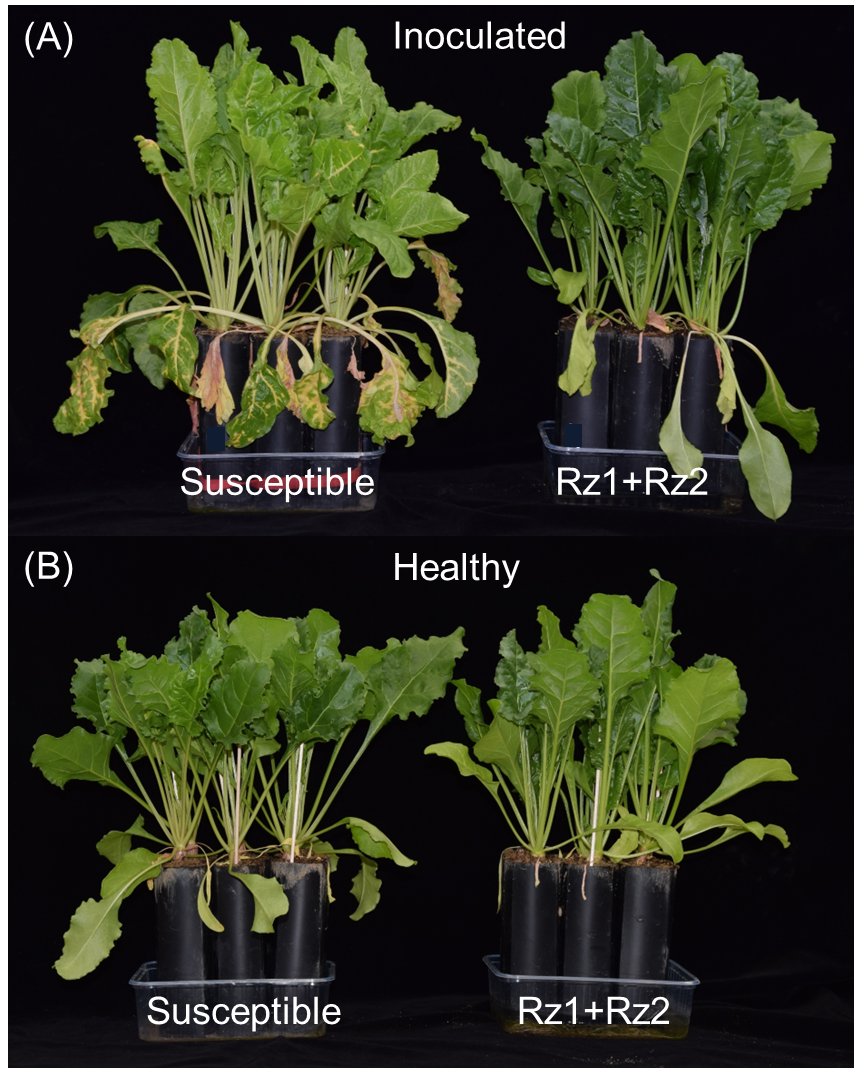
**

Supplementary Figure 4. (A) Sugar beet plants from a susceptible and double resistant genotype (*Rz1*+*Rz2*) inoculated with a BNYVV clone carrying the *Rz1* resistance-breaking tetrad VFHG (6 wpi). (B) Non-inoculated control plants from the susceptible and double resistant genotype (*Rz1*+*Rz2*).
